# The Regulation of Tumor Suppressor p63 by the Ubiquitin-Proteasome System

**DOI:** 10.3390/ijms17122041

**Published:** 2016-12-06

**Authors:** Stephen R. Armstrong, Hong Wu, Benfan Wang, Yasser Abuetabh, Consolato Sergi, Roger P. Leng

**Affiliations:** 1 370 Heritage Medical Research Center, Department of Laboratory Medicine and Pathology, University of Alberta, Edmonton, AB T6G 2S2, Canada; ar2@ualberta.ca (S.R.A.); hwu8@ualberta.ca (H.W.); benfan@ualberta.ca (B.W.); abuetabh@ualberta.ca (Y.A.); 2 Department of Laboratory Medicine and Pathology (5B4. 09), University of Alberta, Edmonton, AB T6G 2B7, Canada; sergi@ualberta.ca

**Keywords:** p63, Mdm2, Mdm4, AIP 4, WWP1, E3 ligases, ubiquitination, degradation

## Abstract

The protein p63 has been identified as a homolog of the tumor suppressor protein p53 and is capable of inducing apoptosis, cell cycle arrest, or senescence. p63 has at least six isoforms, which can be divided into two major groups: the TAp63 variants that contain the N-terminal transactivation domain and the ΔNp63 variants that lack the N-terminal transactivation domain. The TAp63 variants are generally considered to be tumor suppressors involved in activating apoptosis and suppressing metastasis. ΔNp63 variants cannot induce apoptosis but can act as dominant negative inhibitors to block the function of TAp53, TAp73, and TAp63. *p63* is rarely mutated in human tumors and is predominately regulated at the post-translational level by phosphorylation and ubiquitination. This review focuses primarily on regulation of p63 by the ubiquitin E-3 ligase family of enzymes via ubiquitination and proteasome-mediated degradation, and introduces a new key regulator of the p63 protein.

## 1. The p53 Family of Tumor Suppressors

The p53 family of transcription factors is centrally important in cancer research. This family contains the tumor suppressor protein that is most frequently inactivated in cancer, p53, and two other family members, p63 and p73. p53 was discovered in 1979 through SV40 viral transformation studies in animals [1,2,3], during the era when cancer was hypothesized to result from viral transformation. One SV40 antigen, a 53 kDa protein, was thought to be a viral oncogene but was later identified as a host protein [1]. In 1983, the protein was named p53 and was soon after classified not as an oncogene but as a tumor suppressor [1,2]. p53 is inactivated in approximately 80% of all human cancers and mutated in approximately 50% [3,4,5,6,7]. It acts as an important tumor suppressor in response to genotoxic damage [6]. Its tumor suppressor function is thought to increase the expression of transactivating cell cycle arrest proteins (p21) and apoptotic proteins (bax, PUMA) [5,6]. Mutant *p53* in cancer loses the ability to transactivate these proteins [8]. p53 was thought to be the only tumor suppressor in its family until 1997, when two p53 homolog proteins were discovered, p63 and p73 [9,10,11]. p63 was cloned and characterized in 1998 [10,12,13]. Originally, p63 and p73 were assumed to function similarly to p53. However, both p63 and p73 have now been shown to also play developmental roles.

## 2. Structure and Properties of p53 Family Proteins

**Figure 1:**
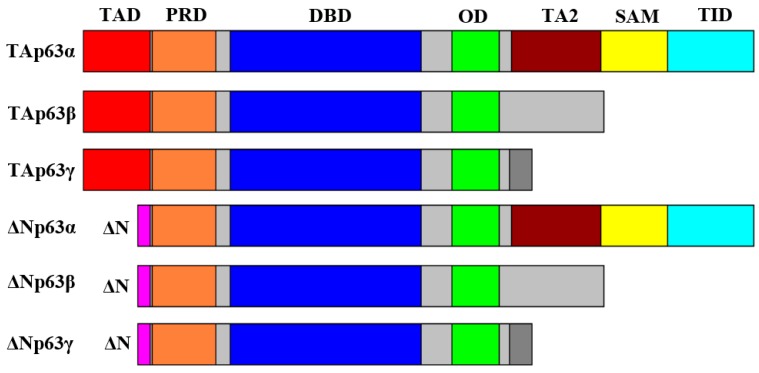
Six isoforms of p63 including all domains of each transcript including the transactivation domain (TAD), the proline-rich domain (PRD), the DNA-binding domain (DBD), the oligomerization domain (OD), the second transactivation domain (TA2), the sterile α motif domain (SAM), and the transactivation-inhibition domain (TID).

p53, p63, and p73 share three domains (Figure 1). An N-terminal acidic transactivation domain (TAD) is responsible for transactivation of target genes. A highly conserved DNA-binding domain (DBD) is responsible for binding to target DNA sequences; the DNA-binding domain has 65% identity between p63 and p53, 62% identity between p73 and p53, and 91% identity between p63 and p73 [14]. An oligomerization domain (OD) is responsible for protein oligomerization into active tetrameric forms [2,3,14,15]. The genes encoding *p63* and *p73* (*TP63* and *TP73*, respectively) [2] have unique properties compared to the *p53* gene (*TP53*). *TP63* and *TP73* contain both a primary promoter (P1) upstream of the coding sequence and an alternative promoter (P2) located in intron 3 [2,13,15]. Unlike *TP63* and *TP73*, the P2 promoter of *TP53* is located in intron 4 and is able to generate ∆133p53, which lacks the transactivation domain and part of the DNA-binding domain [2,16]. The P2 promoter regulates expression of ∆133p53 and ∆160p53. The ∆133p53 isoform stimulates tumor angiogenesis and progression [16].

The P1 and P2 promoters of *TP63* and *TP73* encode two strikingly different groups of isoforms: P1 encodes p53-like isoforms that contain the N-terminal acidic transactivation domain and are designated TAp63/TAp73, while P2 generates N-terminal truncated isoforms that lack this transactivation domain and are designated ΔNp63/ΔNp73 [13,15,17]. The N-terminal transactivation domain is thought to be important for p53-like tumor suppressor functions. Both the TAp63 and TAp73 isoforms are able to transactivate p53-responsive genes related to tumor suppression, such as p21 and bax [13,15]. Although the ΔNp63 and ΔNp73 variants lack the N-terminal transactivation domain [13,15], they are able to transactivate other targets through the function of a second transactivation transactivation domain [18,19].

Further, both p63 and p73 have isoforms that undergo alternative splicing at the C-termini of exons 10–14, producing at least three variants for p63 (Figure 1) and at least nine for p73 [2,9,13,15]. Between the alternative promoters and the splicing variants, p63 has at least six isoforms (TAp63α (full length), TAp63β, TAp63γ, ΔNp63α, ΔNp63β, ΔNp63γ) [2,5,13,15,20] and p73 has at least eighteen isoforms [15]. The α variants of both p63 and p73 contain a sterile α motif domain (SAM) (Figure 1), which is thought to be responsible for protein–protein interactions [2,3,6] and is involved in development [16]. A proline-rich domain (PRD) is present on all p63 isoforms (Figure 1) and is necessary for TAp63β’s transactivation activity and for its ability to mediate apoptosis [21]. α variants of p63 also contain a second transactivation domain (TA2), encoded by exons 11 and 12, that occurs just before the sterile α motif domain [18]. All ΔNp63 variants contain a third transactivation domain at the N-terminus. These additional transactivation domains confer the ability of ΔNp63 variants to transactivate target genes, such as *p21* and *GADD45*, two mediators of cell cycle arrest [19].

## 3. Expression and Functions of p53 Family Proteins

All three members of the p53 family, p53, p63, and p73, arise from a *p63*/*p73* ancestor gene found in almost all invertebrates. This gene was duplicated during the evolution of early vertebrates to produce the *p53* gene, which is primarily a tumor suppressor that controls the cell cycle and apoptosis. The *p63*/*p73* ancestor gene was later duplicated again during the evolution of bony fish to produce the *p63* and *p73* genes, which shared function with p53 but also became specialized in developmental roles [22]. Unlike p53, which is expressed in all cells, p63 and p73 are specifically expressed in epithelial tissues of the ectoderm [23]. p63 is involved in epithelial development [24,25], cell metabolism [26,27], and senescence [27]; p73 is involved in neurogenesis, pheromone signaling, and cerebrospinal fluid dynamics [15,28,29].

**Figure 2:**
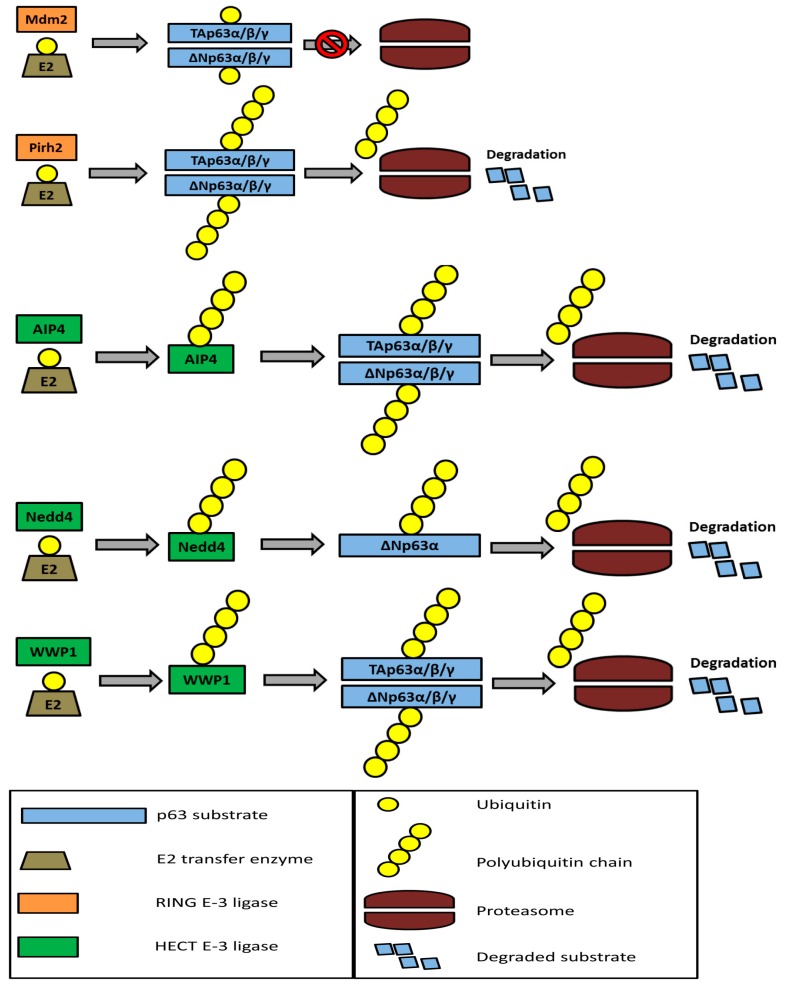
Overview of the regulation of p63 isoforms. This figure outlines the different ubiquitin E3-ligases regulate p63 isoforms and how they interact with the p63 isoforms and subsequent proteasome degradation.

Conflicting expression and functional data cast doubt on whether these two proteins (particularly p63) function as p53-like tumor suppressors or as oncogenes. *p63* rarely undergoes loss of heterozygosity or mutation [6,30]. In fact, the chromosome on which the *p63* gene is located (3q27–29 [13]) is frequently amplified in various cancers, including lung cancer and squamous cell carcinomas of the head and neck [31,32,33], suggesting an oncogenic role [15,34] (Figure 2). *p73* is located on a chromosome region 1p36 [9] that is frequently amplified in various cancers, including breast and colorectal carcinomas [2]. ΔNp63 variants are often overexpressed in cancers of urinary bladder, esophagus, and lung [33,34,35,36,37,38,39]; TAp63 variants are under-expressed in osteosarcomas and carcinomas of the bladder, oral mucosa, and larynx [37,40,41,42], but overexpressed in malignant lymphomas [43].

Unlike mice lacking *p53*, which develop normally and generate spontaneous tumors [35], lack of both *p63* alleles (p63^−/−^) is embryonically lethal, resulting in mice with severe developmental abnormalities including lack of epithelium and anomalies of limb development [24]. Mice lacking both *p73* alleles (p73^−/−^) show neurological abnormalities but no evidence of tumorigenesis [28]. However, when mice lack one allele of either *p63* or *p73* (p63^+/−^ or p73^+/−^), their developmental abnormalities are not severe and they do develop tumors [36]. Furthermore, mice with heterozygous deletions in *p53* and either *p63* or *p73* develop tumors with a greater degree of metastasis than mice with only *p53* heterozygous deletions [36].

ΔNp63 variants are able to inactivate the transactivation function of p53 and variants of both p63 and p73 by directly competing with promoter regions [13,15]. ΔNp63 variants can also inactivate transactivation function by incorporating into heterotetramers and acting in a dominant negative fashion [13,15]. TAp73 can transactivate p53 tumor suppressor targets such as p21 [9], but ΔNp73 is able to inhibit the tumor suppressor functions of p53 and TAp73 through dominant negative heterotetramers or promoter competition [17,28,29,44,45]. TAp73 and p53 both transactivate the expression of ΔNp73, creating a negative feedback loop [44]. Knockout studies of *p53*, *p63*, and *p73* in mouse embryo fibroblasts have demonstrated that p53 requires either TAp63 or TAp73 to induce apoptosis. When both *p63* and *p73* are knocked out together, p53 is unable to induce expression of pro-apoptotic genes, including *bax*, *Noxa*, and *PERP* [46]. On the basis of these observations, TAp63, and TAp73 variants are often thought to function as tumor suppressors and the ΔNp63 and ΔNp73 variants as oncogenes [2,36,46]. The ratios between the TA and ΔN variants of these proteins may be important in determining overall oncogenic or tumor suppressive properties [15,43,47].

The DNA-binding domains of the p53, p63, and p73 transcription factors recognize specific response elements (REs) for binding. p53 recognizes p53 response elements (p53RE) upstream of genes belonging to *bax*, *p21*, *Noxa*, *PUMA*, and many others [48]. p63 can bind to both p53REs and p73REs, but p63 is at least twice as active in transcription when binding to p63REs, which contain different base pairs at the 5th and 16th position compared to the p53RE [49,50]. The specific p63RE sequence is 5′-RRRC**(A/G)**(A/T)GYYYRRRC(A/T)**(C/T)**GYYY-3′ [49,50], with key differences from the p53RE highlighed in bold. Examples of genes that p63 preferentially transactivates to a higher degree than p53 are PTPN14 and ING1, tumor suppressors that are involved in promoting apoptosis, cell cycle arrest, and senescence [49]. Recent functional experiments have demonstrated that TAp63 specifically transactivates Dicer, Sharp-1, and Maspin, factors involved in suppressing metastasis [51,52,53]. This work strengthens the link between TAp63 isoforms and tumor suppressor function. Since TAp63 isoforms likely have tumor suppression roles, understanding how they and the oncogenic ΔNp63 isoforms are regulated is important.

## 4. Kinases and p63 Phosphorylation

p63 is thought to be regulated predominantly at the protein level [54]. Self-regulation of the TAp63α isoform is mediated through a unique transactivation inhibition domain (Figure 1) that can interact directly with the transactivation domain and form inactive dimers [15,55,56]. This transactivation inhibition domain can be cleaved off by caspases 3, 6, 7, and 8 during apoptosis, enhancing TAp63α’s transactivation ability [57]. ΔNp63 variants can inactivate TAp63 variants by either direct promoter competition, or by acting in a dominant negative fashion by forming heterotetramers with TAp63 [13]. Mutant p53 variants are frequently thought to function in a dominant negative function towards TAp63 [58,59] and TAp73 [60] in a similar fashion as ΔNp63. p63 is commonly regulated by post-translational modification, including phosphorylation by various kinases to activate p63 variants. Kinases that activate TAp63 variants include c-Abl [61], Cables1 [62], IKKβ [63], Pin1 [64], PML [65], and TLR3 [66]. Kinases, such as PlK1 [67], inhibit TAp63 variants by phosphorylation of Ser52 in the transactivation domain. Kinases that activate ΔNp63 variants include c-Abl [68], Pin1 [64], and PML [65], while kinases that inhibit ΔNp63 variants include ATM [69], CDK2 [69], HIPK2 [70], p38 [71], p70s6K [69], and Raf1 [72].

## 5. The Ubiquitin-Proteasome System and p63 Regulation

Ubiquitination is another common pathway for p63 regulation, usually via negative regulation of p63 isoforms through the ubiquitin-proteasome system. This system is a major pathway for regulating the cellular proteome by targeting specific proteins for proteasome-mediated degradation [73]. The proteasome is a large multi-subunit protein with a 20S core complex responsible for proteolysis and a 19S regulatory complex responsible for protein recognition [73]. To be recognized for degradation by the proteasome, substrates must first be tagged with ubiquitin, a small 8.5 kDa protein [73,74]. This post-translational modification is carried out by three classes of enzymes designated E-1, E-2, and E-3. E-1 activation enzymes activate ubiquitin in an ATP-dependent manner, attaching it to a cysteine residue of an E-2 conjugation enzyme. The E-2 conjugation enzyme acts in concert with an E-3 ligase enzyme in order to attach ubiquitin to a lysine residue of a target substrate [73,75].

E-3 ligases are substrate-specific [73] and number in the hundreds [76], but can be subdivided into different classes depending on their catalytic domains. RING (really interesting new gene)-type E-3 ligases act as a scaffold, binding to the E2 enzyme and target substrate and bringing them into close proximity. The RING domain catalyzes direct attachment of ubiquitin from the E-2 enzyme to the target substrate in this way [75,77]. U-box domains are structurally similar to RING domains, and function in the same way, but are stabilized by hydrogen bonding rather than zinc ion coordination [78,79]. HECT (homologous to E6-AP carboxyl terminus)-type E-3 ligases act differently, functioning as a catalytic intermediate in transfer of ubiquitin from the E-2 enzyme onto a cysteine residue of the HECT E-3 ligase and then directly to a lysine residue of the target substrate [75,77].

Target substrates can be mono-ubiquitinated (one ubiquitin molecule attached), multi-ubiquitinated (multiple ubiquitin molecules attached to different regions of the substrate) or poly-ubiquitinated (a ubiquitin chain of multiple ubiquitin molecules attached to the substrate [80]). Mono-ubiquitination is involved in endocytosis, membrane trafficking, and subcellular localization [80,81,82], while poly-ubiquitination is involved in protein degradation. Poly-ubiquitination links ubiquitin chains through the ubiqutin lysine residues K6, K11, K27, K29, K33, K48, and K63 [80]. The proteosome canonically recognizes K48-linked poly-ubiquitin chains that are at least four ubiquitin proteins long [83], although K6-, K11-, K27-, and K29-linked chains have also been implicated in proteasome degradation [84]. Poly-ubiquitination requires participation of an E-4 enzyme responsible for facilitating ubiquitin chain elongation [73,75], although some E-3 ligases such as those that contain U-box domains also possess E-4 function [85,86].

Multiple E-3 ligases are responsible for regulating the p63 protein. Mdm2 (murine double minute-2), the most well-known, is an E-3 ligase containing the RING domain that can target p53 for degradation [87] in cooperation with an E-4 enzyme UBE4B [88]. Mdm2 can also mono-ubiquitinate p63 and p73 but is unable to cause degradation of either protein [89,90,91,92]. Its interaction with p63 and p73 is capable of interfering with their transactivation function, likely by exporting them from the nucleus into the cytoplasm [90]. However, the literature contains some disagreement: one study found Mdm2 unable to inhibit p63 function [91], another found that Mdm2 actually stabilized p63, increasing both its expression and its function [93], while yet another found no interaction between Mdm2 and p63 [94]. Mdm2 is able to bind to p53, p63, and p73 through an FxxθxxL sequence (where θ is leucine or isoleucine and x is any amino acid) located in the transactivation domain [92]. Further, both p53 [95] and TAp73 [15] are able to transactivate Mdm2, providing a negative feedback loop for their own expression. Although Mdm2 is incapable of targeting p63 for degradation, it can cooperate with Fbw7 (an F-box ligase) to poly-ubiquitinate ΔNp63 and target it for proteasome degradation [89]. MdmX is an E-3 ligase related to Mdm2, but it does not have the ability to target any of the p53 family members for degradation and cannot interfere with p63 or p73 function [90,91].

Pirh2 (p53-induced protein with an RING-H2 domain) [96] is a RING-containing E-3 ligase able to bind to and target all members of the p53 family for degradation [97,98,99,100]. Pirh2 is able to induce degradation of both TAp63 and ΔNp63 isoforms [99], in cooperation with the E-2 enzyme UbcH5b [97]. Pirh2 can also induce degradation of p73 [99,101], and p21 [86]. It can be transactivated by p53, another example of a negative feedback loop and possible competition between family members [96].

Itch/AIP4 (atrophin-1 interacting protein 4) [102] is a HECT E-3 ligase that can target both p63 and p73 for proteasome degradation [103,104]. It is considered the major regulator of p63 protein, able to target all isoforms for proteasome degradation [102,104,105]. Itch/AIP4 functions as a monomer with four WW protein–protein interaction domains and a C-terminal HECT domain [102]. It requires its HECT domain for ubiquitination [104]. The WW domains recognize the PY motif (a short proline-rich segment PPPXY) in the proline-rich domain of p63 and p73, which is located between the transactivation and DNA-binding domains [102]. Phosphorylation of threonine on this motif is crucial for WW interaction and subsequent ubiquitination by Itch/AIP4 [106].

Nedd4 is a HECT-containing E-3 ligase able to bind to ΔNp63, ubiquitinate it, and target it for degradation [107]. It binds ΔNp63α, but not ΔNp63γ, recognizing a PY motif on ΔNp63α’s sterile α motif domain. Nedd4 contains three central WW domains, in addition to the C-terminal HECT domain, which are likely responsible for recognizing the PY motif on ΔNp63α [107]. Although TAp63α also contains the sterile α motif domain, the literature contains no mention of Nedd4 being able to target TAp63α for degradation, and Nedd4 may be specific to the oncogenic α isoforms of the p63 protein.

WWP1 is a HECT-containing E-3 ligase targeting both TAp63 and ΔNp63 for proteasome degradation. Similarly to Itch/AIP4, it binds to the PY motif on those proteins using its WW domains and ubiquitinates them using its HECT domain. WWP1 has both tumor suppression and oncogenic roles that are thought to depend on the context of the cell line in which it is expressed. Knockdown of WWP1 in the breast cancer 184B5 cell line is associated with a decrease of ΔNp63 levels, while knockdown of WWP1 in colorectal HCT116 cells increases TAp63 expression and sensitivity to genotoxic stress [108]. The regulation of p63 by several ubiquitin E3 ligases is summarized in Figure 2.

## 6. Summary

p63 is a highly complex set of proteins with isoform-dependent functions ranging from development to tumor suppression to tumor promotion. Both TAp63 and ΔNp63 are tightly regulated at the protein level. Multiple E-3 ligases control their protein levels, including RING-containing and HECT-containing E-3 ligases. Some E-3 ligases are isoform specific, while others can only target certain p63 splicing variants for degradation. Like p63 itself, these E-3 ligases are often seen as oncogenic or tumor-suppressive depending on which isoforms they target, and their roles may be context dependent.
